# P-1132. Patient Pre-Selection Improves Efficiency and Acceptability of Antimicrobial Audit-and-Feedback Rounds in a Neonatal Intensive Care Unit

**DOI:** 10.1093/ofid/ofae631.1319

**Published:** 2025-01-29

**Authors:** Marie-Astrid Lefebvre, Gabrielle Girard, Christos Karatzios, Earl Rubin, Jane McDonald

**Affiliations:** Montreal Children's Hospital, McGill University Health Centre, Montreal, Quebec, Canada; Montreal Children's Hospital, McGill University Health Centre, Montreal, Quebec, Canada; Montreal Children's Hospital, McGill University Health Centre, Montreal, Quebec, Canada; Montreal Children's Hospital, McGill University Health Centre, Montreal, Quebec, Canada; Montreal Children's Hospital, McGill University Health Centre, Montreal, Quebec, Canada

## Abstract

**Background:**

Audit-and-Feedback Rounds (AFR) are an effective way of evaluating the appropriateness of antimicrobials in inpatient settings, but they are time- and resource-intensive. In our 52-bed tertiary care neonatal intensive care unit (NICU), AFR methodology was modified in that pharmacists would pre-select patients to be discussed, in order to focus on value-added discussions. This observational study describes the impact of this intervention on key AFR metrics.

**Figure 1.**
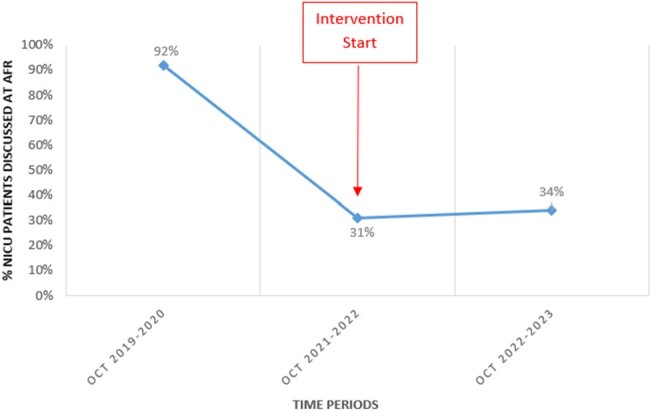
Percentage of NICU patients on systemic antimicrobials who were discussed at audit-and-feedback rounds, pre- and post-intervention.

**Methods:**

Since October 2019, the NICU team and an Infectious Diseases (ID) physician met weekly to review all patients on systemic antimicrobials at the time of AFR. The ID physician assessed the appropriateness of antimicrobials and made recommendations to the team. Pharmacists prospectively collected data on the duration of AFR, reasons for inappropriate prescriptions, recommendations made, and adherence to recommendations 24 hours later. To improve efficiency, from September 2021 on, it was agreed that pharmacists would select out some patients from the discussion list as they were considered by default to be on appropriate therapy: those followed by the ID service; neonates on empiric ampicillin and an aminoglycoside for early-onset sepsis; and those on prophylactic antimicrobials if already reviewed once before.

**Figure 2.**
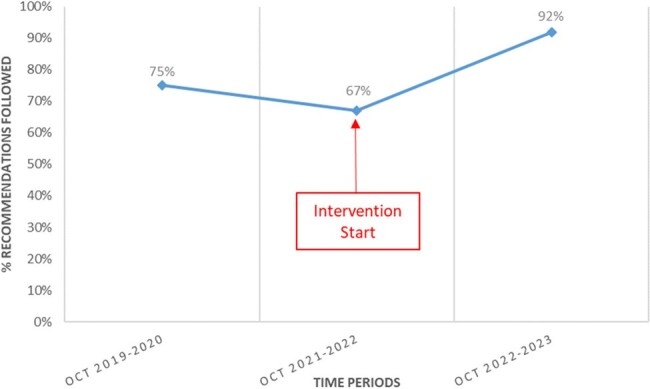
Percentage of antimicrobial stewardship recommendations accepted by NICU team, assessed 24 hours post-rounds.

**Results:**

In the pre-intervention period (Oct 2019 - Oct 2020), 92% (n = 226) of patients on systemic antimicrobials were reviewed, and AFR lasted 27 minutes on average. Antimicrobial use was considered appropriate for 88% of patients and 75% (n=55) of recommendations were accepted by the NICU team. In the initial post-intervention period (Oct 2021- Oct 2022), 30 % (n = 101) of patients on antimicrobials were discussed at AFR (Figure 1), and usage was considered appropriate in 80% (n = 123). Between Oct 2022 and Oct 2023, mean duration of AFR had decreased to 13 minutes, and adherence to recommendations increased to 92% (n=36).

**Conclusion:**

Patient pre-selection for AFR was associated with shorter rounds and increased uptake of recommendations by clinical teams. This method could be used to conduct AFR in other busy inpatient settings.

**Disclosures:**

**All Authors**: No reported disclosures

